# Health Outcomes of Discontinuing Antipsychotics After Hospitalization in Older Adults

**DOI:** 10.1093/geroni/igaf122.359

**Published:** 2025-12-31

**Authors:** Dae Hyun Kim, Chun-Ting Yang, James Wilkins, Elyse DiCesare, Kevin Pritchard, Qiaoxi Chen, Yichi Zhang, Joshua Lin

**Affiliations:** Hebrew SeniorLife, Boston, Massachusetts, United States; Brigham and Women’s Hospital, Boston, Massachusetts, United States; McLean Hospital, Belmont, Massachusetts, United States; Brigham and Women’s Hospital, Boston, Massachusetts, United States; Brigham and Women’s Hospital, Boston, Massachusetts, United States; Brigham and Women’s Hospital, Boston, Massachusetts, United States; Brigham and Women’s Hospital, Boston, Massachusetts, United States; Brigham and Women’s Hospital, Boston, Massachusetts, United States

## Abstract

Antipsychotic medications (APMs) prescribed to manage behavioral symptoms of delirium during hospitalization are often continued after discharge. Whether discontinuing APM post-discharge is associated with better clinical outcomes is unknown. This retrospective cohort study utilized Medicare 2013-2018 and Optum Clinformatics® 2004-2024 to examine the clinical outcomes of discontinuing versus continuing APMs following hospitalization in older adults. Patients aged≥65 years without psychiatric disorders or prior APM use who filled an APM prescription within 30 days of hospital discharge were included. APM discontinuation was defined as having a dispensing gap of more than 45 days following the end of an APM prescription. We used an incidence density sampling approach to select APM continuers, matching the type of APM prescribed, time since the first APM prescription, and intensive care unit admission as the discontinuers. Propensity score matching was applied to balance baseline covariates. Study outcomes included all-cause rehospitalization, specific rehospitalization reasons, and all-cause mortality. Hazard ratio (HR) comparing APM discontinuation versus continuation was estimated using Cox proportional hazards model. Estimates from the two databases were pooled by inverse variance weighting. A total of 13,955 propensity score-matched pairs were identified. Over the median follow-up of 180 days, APM discontinuers showed significantly lower risks of rehospitalization (HR: 0.88 [0.84-0.93]), inpatient delirium (0.87 [0.79-0.96]), fall-related emergency visits or hospitalizations (0.80 [0.69-0.93]), and all-cause mortality (0.78 [0.70-0.88]) compared with the continuers. Non-significant reductions were observed for pneumonia (0.95 [0.78-1.15]) and urinary tract infections (0.85 [0.72-1.02]). These findings suggest the importance of minimizing duration of APM use after discharge.

